# Exploiting Complex Fluorophore Interactions to Monitor Virus Capsid Disassembly

**DOI:** 10.3390/molecules26195750

**Published:** 2021-09-22

**Authors:** Swarupa Chatterjee, Bram A. Schotpoort, Thieme Elbert, Jeroen J. L. M. Cornelissen, Mireille M. A. E. Claessens, Christian Blum

**Affiliations:** 1 Nanobiophysics (NBP), MESA + Institute for Nanotechnology and Technical Medical Centre, Faculty of Science and Technology, University of Twente, P.O. Box 217, 7500 AE Enschede, The Netherlands; s.chatterjee-1@utwente.nl (S.C.); b.a.schotpoort@student.utwente.nl (B.A.S.); t.elbert@student.utwente.nl (T.E.); m.m.a.e.claessens@utwente.nl (M.M.A.E.C.); 2 Wetsus, European Centre of Excellence for Sustainable Water Technology, 8911 MA Leeuwarden, The Netherlands; 3 Biomolecular Nanotechnology (BNT), MESA + Institute for Nanotechnology, Faculty of Science and Technology, University of Twente, P.O. Box 217, 7500 AE Enschede, The Netherlands; j.j.l.m.cornelissen@utwente.nl

**Keywords:** Förster Resonance Energy Transfer, photophysical interactions, fluorophore self-quenching, dark aggregates, virus capsid, virus inactivation

## Abstract

Supramolecular protein complexes are the corner stone of biological processes; they are essential for many biological functions. Unraveling the interactions responsible for the (dis)assembly of these complexes is required to understand nature and to exploit such systems in future applications. Virus capsids are well-defined assemblies of hundreds of proteins and form the outer shell of non-enveloped viruses. Due to their potential as a drug carriers or nano-reactors and the need for virus inactivation strategies, assessing the intactness of virus capsids is of great interest. Current methods to evaluate the (dis)assembly of these protein assemblies are experimentally demanding in terms of instrumentation, expertise and time. Here we investigate a new strategy to monitor the disassembly of fluorescently labeled virus capsids. To monitor surfactant-induced capsid disassembly, we exploit the complex photophysical interplay between multiple fluorophores conjugated to capsid proteins. The disassembly of the capsid changes the photophysical interactions between the fluorophores, and this can be spectrally monitored. The presented data show that this low complexity method can be used to study and monitor the disassembly of supramolecular protein complexes like virus capsids. However, the range of labeling densities that is suitable for this assay is surprisingly narrow.

## 1. Introduction

Fluorescence microscopy and spectroscopy techniques are indispensable in soft matter and life science research. Fluorescent labeling, for example, allows for the localization of cellular components in fluorescence microscopy and for the quantification of concentrations in fluorescence spectroscopy approaches [1,2,3,4,5,6]. Environment-sensitive fluorophores are used to report on the polarity of the environment, the pH or the presence of specific ions [7,8,9]. To probe (bi)molecular interactions, Förster Resonance Energy Transfer (FRET) is one of the most used methods [10,11,12]. In FRET assays, interactions are monitored by the change in fluorescence properties when FRET donor and acceptor dyes that are conjugated to the two interaction partners of interests come in nanometer proximity of each other. However, in a biological context, interactions are often not limited to two interaction partners, as many multi-component and self-assembled systems containing a large number of (macro)molecules can be found in the cell [13]. How such larger systems self-assemble and disassemble is an intriguing question.

One especially interesting example of a multicomponent self-assembled biosystem is the virus capsid [14,15]. The capsid is a self-assembled shell composed of hundreds of proteins that encloses the genetic material of the virus [16]. In non-enveloped viruses, the capsid is in direct contact with the environment and necessary for cell penetration. For these viruses to be functional, the capsid has to be intact. Methods that monitor capsid (dis)assembly thus report on the functional state of the virus. Such monitoring is of interest not only for testing inactivation strategies and new disinfectants, but also because viruses are used as biomolecular platforms, e.g., as nano-carriers [17,18,19] or nano-reactors. [20] For these applications, the possibility to track the functional status of the virus by monitoring the capsid integrity in cells would be beneficial. Virus localization often does not provide enough information for optimization strategies as cargo release depends on capsid breakdown.

Current methods to monitor the breakdown of self-assembled complexes of bio(macro)molecules include Fluorescence Correlation Spectroscopy (FCS), Dynamic Light Scattering (DLS), NMR and the monitoring of the presence of binding epitopes for antibodies only present in assembled viruses [21,22,23]. Most of these methods require high end equipment, expertise, and they are laborious. Moreover, both DLS and NMR require a lot of material and are not suitable for in vivo measurements at the single cell level. For viruses, RT-PCR and cell cultures are used to monitor infections and serve as semi-quantitative measures of their presence [24]. However, although effective, these methods are time and labor intensive and not very well suited for studies towards functional goals including the design of new disinfectants, nano-carriers and the optimization of virus removal membranes [25,26,27]. There is a need for new, fast and easy methods to assess the functional state of viruses. Fluorescence-based methods are very sensitive, of low experimental complexity and can be used both in vitro and in vivo.

Here, we investigate a new strategy to monitor the disassembly of fluorescently labeled viruses. To test this strategy, we make use of a fluorescently labeled plant virus as a model for non-enveloped viruses. To disassemble these viruses, we use the anionic surfactant Sodium Dodecyl Sulfate (SDS). To monitor disassembly, we exploit the complex photophysical interplay between multiple fluorophores conjugated to proteins of the virus capsid. The disassembly of the capsids changes the photophysical interactions between the fluorophores, and this can be spectrally monitored. The presented data show that the assay can be used to report on the functional state of the capsid. However, the range of labeling densities that is suitable for this assay is surprisingly narrow. Disentangling the photophysical effects, and thus discriminating between breaking fluorophore interactions and protein–protein interactions, remains challenging.

## 2. Results and Discussion

**Figure 1:**
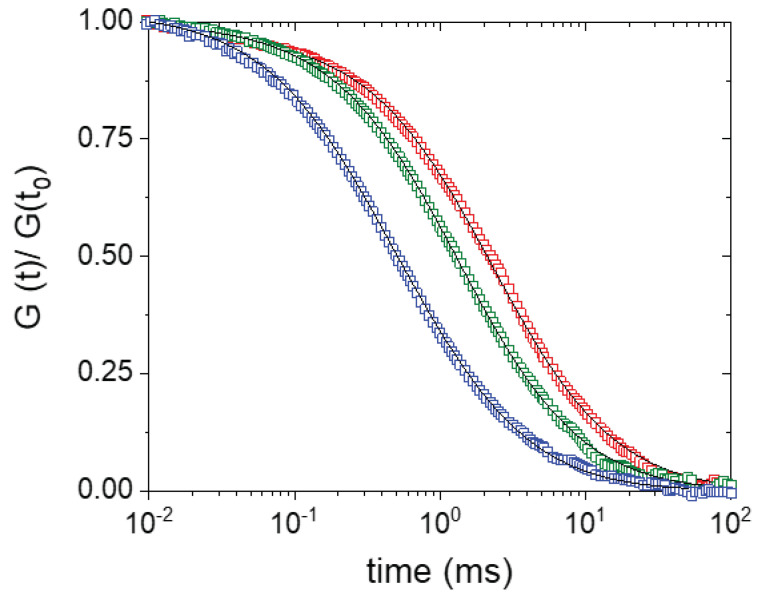
SDS-induced CCMV capsid disassembly monitored with FCS. FCS autocorrelation curves of Atto647N-labeled viruses in the absence of SDS (red), in the presence of 0.35 mM SDS (green) and in the presence of 1.7 mM SDS (blue) in 50 mM phosphate buffer (pH 7.4). The experimental data are shown in open symbols, the fit to the data is shown as a line. The data were normalized to G(t) at 10 µs. Capsid disassembly is visible as a shift to lower correlation times.

The addition of surfactants is known to result in the disassembly of both enveloped and non-enveloped viruses [21,22,28]. To verify that the surfactant Sodium Dodecyl Sulfate (SDS) can be used to disassemble the capsid of the non-enveloped Cowpea Chlorotic Mottle Virus (CCMV), we directly monitored capsid disassembly in a Fluorescence Correlation Spectroscopy (FCS) experiment. We excited Atto647N-labeled CCMV virus capsids and monitored their diffusion through the detection volume. By autocorrelating the resulting fluorescence intensity fluctuations, typical FCS curves were obtained. In Figure 1, we present the FCS curves for three different SDS concentrations. In the absence of SDS, the autocorrelation curve can be fitted with single diffusion coefficient, which corresponds with the presence of spherical particles with a diameter of 27.2 nm and matches the diameter of intact CCMV. With the increasing SDS concentration, the FCS curves shift to lower times corresponding to an increase in the diffusion coefficient. At 0.35 mM SDS, we observed an intermediate diffusion coefficient, most likely representing partially disassembled virus capsids. At 1.7 mM SDS, the diffusion coefficient was determined to be ~64 µm^2^/s, which corresponds to a spherical particle of approximately 7 nm in diameter. With increasing SDS concentration, we did not observe a further decrease of the correlation time. The diameter of a single capsid protein is estimated from its molecular weight to be approximately 4.3 nm, and SDS micelles typically have a diameter of ~5 nm. The FCS data indicate that the viruses most likely disassembled into monomers/dimers, which are solubilized by SDS. The presence of both SDS and capsid proteins would account for the relatively large size. Concluding, the FCS data show that at 1.7 mM SDS the CCMV capsid disassembled completely; in further CCMV disassembly studies, we used this concentration as an upper bound.

While FCS is a useful technique to study the disassembly of fluorescently labeled capsids, the method is complex and requires specialized instrumentation. A spectral readout to study virus disassembly would be much easier and faster. A way to realize such an assay makes use of the fact that fluorophores that are in close proximity of each other strongly interact. When antibodies or viruses are labeled with multiple fluorophores, this strong interaction results in less bright particles than expected [29,30,31]. The close proximity of fluorophores results in self-quenching of the fluorescence, which limits the brightness that can be achieved [32]. Fluorophore interactions that are responsible for self-quenching include the formation of dark fluorophore aggregates and the transfer of excitation energy to these dark aggregates [15,33,34,35]. Self-quenching of fluorescence not only manifests as a lower particle brightness than expected, but it additionally results in a shift of the emission peak to higher wavelengths [36]. Although generally a nuisance in labeling strategies, self-quenching may be exploited in applications. For self-quenching to occur, the fluorophores do not have to be on the same protein, it is also observed in protein assemblies like viruses [36]. When the total number of fluorophores on the protein assemblies is much smaller than the total number of virus proteins, individual proteins typically contain less than one fluorophore. This implies that it should be possible to study self-assembly (and disassembly) of labeled protein complexes using the photophysical behavior of the labels.

**Figure 2:**
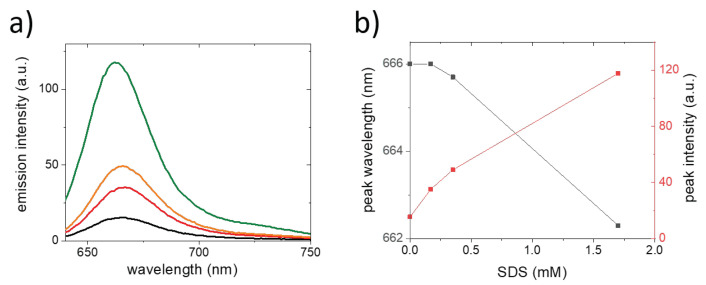
SDS-induced CCMV capsid disassembly monitored using changes in the emission spectrum. The data shown were acquired for CCMV labeled with Atto647N at a DOL of 43 fluorophores/virus in 50 mM phosphate buffer (pH 7.4). The spectra were obtained 30 min after the addition of SDS. (**a**) Emission spectra obtained at no SDS (black), 0.17 mM SDS (red), 0.35 mM SDS (orange) and 1.7 mM SDS (green). (**b**) With increasing SDS concentration, the emission intensity strongly increases (red), while the peak wavelength shifts to shorter wavelengths at high concentrations (black).

To test this approach, CCMV capsids were labeled with an average of 43 fluorophores per virus, which is less than 1 fluorophore per capsid protein. For the intact, assembled and labeled capsids, we observed a moderately bright emission of Atto647N with a peak at a wavelength of 666 nm. Upon the addition of increasing concentrations of SDS, we observed a strong dequenching of the fluorescence and a shift of the emission peak to lower wavelengths (Figure 2). Virus disassembly clearly results in fluorescence dequenching. Quenching of fluorescence due to possible interactions of fluorophores with released RNA only plays a minor role in the observed changes in fluorescence intensity. This indicates that self-quenching at high labeling densities can indeed be used to study the disassembly of protein assemblies. The high degree of labeling may, however, affect the stability of protein assemblies and thereby limit the applicability of this method. Additionally, the spectral shifts observed upon virus capsid disassembly are subtle and the fluorescence intensity is a difficult parameter since it also depends on the sample concentration. Instead of just relying on one fluorophore species, an approach that makes use of fluorophores that form FRET pairs may be better suited since it will require lower labeling densities.

**Figure 3:**
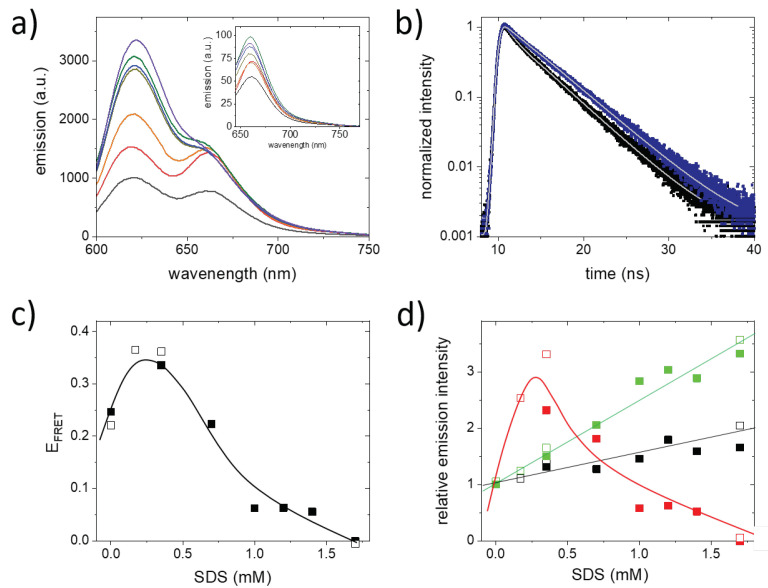
SDS-induced capsid disassembly of CCMV labeled with the FRET pair of Atto590 and Atto647N. (**a**) Emission spectra obtained for CCMV capsids at a DOL of 21 fluorophores/virus and a donor to acceptor ratio of 14:7 in 50 mM phosphate buffer (pH 7.4). Data are shown in the absence of SDS (black), and in the presence of 0.35 mM (red), 0.7 mM (orange), 1.7 mM (yellow), 1.2 mM (green), 1.4 mM (blue) and 1.7 mM (violet) SDS. The main figure shows the emission spectra upon excitation of Atto590 (FRET donor), and the inset shows the emission spectra upon excitation of Atto647N (direct FRET acceptor excitation). (**b**) Peak normalized fluorescence decays of the FRET donor Atto590. Decays were recorded for intact CCMV (0 SDS, black) and disassembled CCMV (1.7 mM SDS, blue). Data are shown as filled symbols, the fit to the data is visible as a gray line. (**c**) E_FRET_ estimated from the donor and acceptor intensity taking into account the contribution of the donor at the peak of the acceptor emission. Data are shown for capsids with a DOL 21 fluorophores/virus and a donor to acceptor ratio of 14:7 (filled symbols) and with a DOL 14 fluorophores/virus at a donor to acceptor ratio of 10:4 (open symbols). The line serves as a guide to the eye. (**d**) Emission intensities as a function of the SDS concentration normalized to the emission intensity in the absence of SDS. The donor emission intensity is shown in green, the acceptor emission intensity upon direct excitation in black and the acceptor emission intensity considering the contribution of the donor at the peak of the acceptor emission is shown in red. Data are shown for capsids with DOL 21 fluorophores/virus at a donor to acceptor ratio of 14:7 (filled symbols) and with DOL 14 fluorophores/virus at a donor to acceptor ratio of 10:4 (open symbols). The lines serve as a guide to the eye.

**Figure 4:**
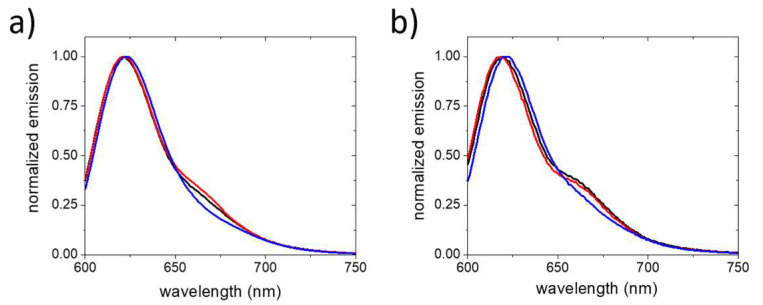
Peak normalized emission spectra of FRET-labeled CCMV in the absence of SDS (black), at 0.35 mM SDS (red) and 1.7 mM SDS (blue) in 50 mM phosphate buffer (pH 7.4). (**a**) CCMV with a DOL of 26 fluorophores/virus and a donor to acceptor ratio of 21:5; (**b**) CCMV with a DOL of 8 fluorophores/virus of and a donor to acceptor ratio of 5:3.

To verify this idea, we labeled CCMV capsids with Atto590 as FRET donor fluorophores and Atto647N as FRET acceptor fluorophores with a total Degree Of Labeling (DOL) between approximately 8 to 26 fluorophores per virus and donor to acceptor ratios between 1:1 and 4:1. Surprisingly, we only observed clear signs of FRET in a limited number of samples. At overall low DOL (<10), we did not observe clear signs of FRET (for examples, see Figure 4). In these samples, the mean distance between the fluorophores was probably too large for FRET to occur. At high labeling densities, we also observed no clear signs of FRET. For DOLs of 21 and 14 fluorophores per virus and donor to acceptor ratios of 14:7 and 10:4, respectively, clear signatures of FRET could be seen. In the emission spectra, both the donor and acceptor peak were visible after donor excitation and the peaks appeared at the expected wavelengths of 622 nm and 662 nm. To assess if we could follow SDS-induced virus capsid disassembly using a FRET assay, we first selected the DOL 21 sample. Upon the addition of SDS, we observed a complex evolution of both the donor and acceptor fluorescence intensities (Figure 3a). The donor intensity increased with the SDS concentration. This was expected, and upon disassembly, the population of FRET-coupled fluorophores decreased, and the average distance between the donor and acceptor dye should increase. With the increased average distance between the donor and acceptor fluorophores, the apparent FRET efficiency should drop, which is visible as an increase in the emission intensity of the donor. However, the acceptor emission intensity initially also increases. This is unexpected, since in a FRET system, an increase in donor emission should coincide with a complementary decrease in acceptor fluorescence. The signature of FRET is also visible in the fluorescence decay of the energy transfer donor fluorophore. We hence recorded fluorescence decays of the FRET-labeled virus sample in the presence of 0 and 1.7 mM SDS (Figure 3b). For all FRET-labeled samples we observed the same behavior. At 1.7 mM SDS, the fluorescence decay followed a single exponential with decay time of approximately 4.3 ns. For the intact viruses, in the absence of SDS, we observed a strongly non-mono-exponential decay, which can be approximated by a double-exponential decay with decay components of <1 and <4 ns. The fluorescence lifetime of the virus samples at 1.7 mM SDS was slightly larger than the lifetime of free non-protein-bound Atto590. The increase in the fluorescence lifetime compared to the free dye may be caused by the presence of SDS. The mono-exponential fluorescence decay at 1.7 mM SDS confirmed that the capsid was fully disassembled. The non-mono-exponential decay in the absence of SDS is the result of different quenching processes. This is reflected by the observation that the contributions to the double exponential decay vary between samples with a different DOL and donor to acceptor ratio.

The initial increase in the acceptor emission intensity upon the addition of SDS indicates that, in the intact virus, the FRET acceptor fluorophores are quenched. The spectral changes observed in the virus disassembly assay are thus not purely the result of changes in FRET, they can probably be partly attributed to a decrease in fluorophore self-quenching. To separate the effect of fluorophore self-quenching from the effect of FRET, we directly excited the acceptor fluorophore (Atto647N) and recorded emission spectra (Figure 3a, inset). In these spectra, fluorophore dequenching is visible as an increase in the emission intensity of Atto647N with increasing SDS concentration. On the intact virus capsid, the formation of dark fluorophore aggregates that serve as energy sinks is the most likely source of fluorescence quenching. However, at DOL 21 with a 14:7 donor to acceptor ratio, a single virus contains on average only eight Atto647N fluorophores; thus, the formation of dark Atto647N aggregates seems unlikely. However, the formation of mixed dark aggregates containing both Atto590 and Atto647N fluorophores may explain the observed strong fluorescence self-quenching.

The contribution of FRET to the signal can be determined by estimating the FRET efficiency (E_FRET_) from the spectral data. In the absence of the donor, the acceptor emission upon excitation at 590 nm is very low. In estimating the E_FRET_, we can therefore neglect direct excitation of the acceptor fluorophore. Both the FCS data and life-time data show that at 1.7 mM the capsid is fully disassembled, and FRET can no longer occur. Moreover, the spectrum obtained at the highest SDS concentration agrees well with the spectrum expected for the Atto590 fluorophore. In the estimation of the E_FRET_, the spectrum obtained at 1.7 mM SDS was therefore used to correct for the donor contribution at the acceptor emission wavelength. The E_FRET_ was subsequently estimated from the corrected data using the following expression:

(1) $$E_{\mathrm{FRET}}=\frac{I_{\mathrm{acceptor}}^{\mathrm{peak}}}{I_{\mathrm{donor}}^{\mathrm{peak}}+I_{\mathrm{accept}}^{\mathrm{peak}}}$$

where $I_{\mathrm{acceptor}}^{\mathrm{peak}}$ is the corrected acceptor peak intensity and $I_{\mathrm{donor}}^{\mathrm{peak}}$ is the donor peak intensity. The evolution of the E_FRET_ as a function of the SDS concentration is plotted in Figure 3c. For the intact virus, in the absence of SDS, the E_FRET_ amounts to approximately 0.25. The addition of 0.35 mM SDS results in an increase of the E_FRET_ to approximately 0.35. At higher SDS concentrations, the E_FRET_ continuously drops. Virus disassembly results in a larger mean distance between the fluorophores. We therefore expected a decrease in FRET with increasing SDS concentration; virus disassembly cannot account for the initial increase of the E_FRET_.

To obtain further insights into the unexpected increase in E_FRET_, we quantified the increase in the relative acceptor fluorescence after both indirect excitation via FRET and direct excitation. Additionally, we plotted the relative change in donor fluorescence (Figure 3d). In samples in which we directly excited the acceptor fluorescence, we observed an almost linear increase in the peak fluorescence intensity with SDS concentration. The same trend was observed for direct excitation of the donor fluorescence. However, the high(er) number of donor fluorophores compared to acceptor fluorophores results in a stronger increase of the donor intensity with the SDS concentration. The evolution of the acceptor peak intensity after indirect FRET excitation gives a very different picture. After increasing to more than double the initial value, the relative acceptor peak intensity sharply decreases with the increasing SDS concentration. The initial increase in E_FRET_ (Figure 3c) and the very strong increase of the acceptor emission after FRET excitation (Figure 3d) show that, at low SDS concentrations, the observed spectral changes are dominated by a decrease in fluorophore self-quenching. The initial increase in both parameters can be attributed to the disappearance of self-quenched dark fluorophore aggregates. By dequenching, more donors become available for energy transfer, and this results in an initial increase in E_FRET_. Initially, at low SDS concentrations, the disappearance of dark fluorophore aggregates contributes more strongly to the spectral changes than the decrease in E_FRET_ due to virus disassembly. At concentrations >1 mM, the effect reverses and the spectral changes mainly result from the progressing virus disassembly.

To test if the photophysical behavior upon disassembly of the labeled viruses changes at different DOLs, we performed experiments at a lower DOL of 14 and a higher donor to acceptor ratio of 10:4. In the emission spectrum of the intact virus, clear indications of FRET are visible. As before, we determined the FRET efficiency and the evolution of both the donor and acceptor intensities with the SDS concentration (Figure 3c,d). The general trend observed in the FRET efficiency and the changes in donor and acceptor fluorescence are comparable to those observed for the DOL 21 sample. However, the changes in E_FRET_ are larger; in the intact virus, E_FRET_ is lower, and upon SDS-induced disassembly, the increase at low SDS concentrations is larger (Figure 3c). This stronger increase is a result of the even stronger dequenching of the FRET-excited acceptor fluorophore (Figure 3d).

Considering that the SDS-induced decrease in fluorophore self-quenching and virus disassembly have opposing effects on the emission spectra of viruses labeled with FRET donor and acceptor fluorophores, large spectral changes may even occur in samples that do not show much FRET in the intact state. To test if this indeed the case, we revisited a high DOL and low DOL FRET-labeled virus sample. For the intact high DOL virus sample, with 26 fluorophores per virus and a donor to acceptor ratio of 21:5, FRET results in a minor contribution of acceptor fluorescence to the emission spectrum. To better visualize spectral changes resulting from the addition of SDS, the emission spectra were normalized to the donor peak emission intensity at 622 nm (Figure 4). In these normalized spectra, the presence of 0.35 mM SDS results in a subtle increase in the acceptor emission at 662 nm. This indicates that, as observed for DOL 14 and 21, the increase in FRET due to fluorophore dequenching still dominates over the decrease in FRET due to virus disassembly. At 1.7 mM, only donor fluorescence can be observed, indicating the total loss of FRET due to virus disassembly. For a low DOL sample with 8 fluorophores/virus and a donor to acceptor fluorophore ratio of 5:3, no spectral changes are observed upon the addition of 0.35mM SDS (Figure 4). At this DOL, the two opposing effects on the emission spectra are balanced and no change in FRET is observed. Increasing the SDS concentration to 1.7 mM again results in the emission spectrum of the donor fluorophore.

Concluding, the presence of multiple fluorophores on one virus capsid results in a myriad of photophysical interactions. Dark fluorophore aggregates and energy transfer processes give rise to a complex spectral response upon SDS-induced disassembly of the virus capsid. For FRET-labeled capsids, the dequenching of both FRET donor and acceptor results in an increase of the E_FRET_ at low SDS concentrations. At higher SDS concentrations, the breaking of FRET interactions between dyes on different proteins dominates, resulting in a sharp drop in FRET efficiency. The effects of these processes on the emission spectra can easily be monitored in a standard spectrophotometer. However, the range of labeling densities that is suitable for spectrally monitoring capsid disassembly is surprisingly narrow.

## 3. Materials and Methods

All chemicals were purchased from Sigma Aldrich unless stated otherwise.

### 3.1. Preparation of Fluorescently Labeled CCMV

The wild-type CCMV virus was obtained following the protocol reported in the literature [37,38]. The crystal structure reported for CCMV confirms that at least six surface-exposed primary amine groups are present per subunit of capsid protein [39,40]. These primary amines are used here to fluorescently label the virus. With a total number of 180 capsid proteins per virus, the maximum number of fluorophores per virus is potentially large. The formation of stable amide bonds between the amine groups on the capsid proteins and fluorophores was performed by following the procedure reported in the literature [41]. In short, the amine groups on the capsid proteins of CCMV were allowed to react with the N-hydroxysuccinimidyl (NHS) ester of Atto647N (ATTO-TEC GmbH) for studies on CCVM labeled with a single fluorophore species. For FRET studies, the CCMV was labeled with the N-hydroxysuccinimidyl (NHS) ester of Atto647N and Atto590 (ATTO-TEC GmbH), which form a FRET pair. The labeling was performed by mixing a range of excess fluorophore concentrations into 400 μL of a 0.43 μM CCMV solution in 50 mM phosphate buffer (pH 7.5). This solution was incubated for 1 h at room temperature. Subsequently, the modified viruses were separated from unreacted fluorophores using a zeba-spin desalting column (30kD molecular weight cut-off) and stored at 4 °C.

### 3.2. Characterization of Fluorescently Labeled CCMV

For the photophysical characterization of the labeled CCMV particles, we made use of several instruments. Absorbance spectra were recorded on a UV–vis spectrophotometer (UV-2600, Shimazu). Fluorescence decays were measured on a spectrofluorometer (FluoroMax 4, Horiba-Jobin, Edison, NJ, USA) equipped with the Time-Correlated Single-Photon Counting (TCSPC) (Coventry, UK) extension. Diffusion constants were measured using fluorescence correlation spectroscopy (FCS) on a PicoQuant MicroTime 200 (Berlin, Germany) confocal microscope and analyzed using the SymphoTime software (Berlin, Germany). The data were fitted assuming a single diffusing species and taking into account triplet formation. All experiments were carried out in 50 mM phosphate buffer (pH 7.4) at room temperature.

The number of attached fluorophore molecules per virus (or degree of labeling, DOL) was derived from the absorbance at 260 nm, 594 nm and 646 nm for single-labeled and FRET-labeled samples. CCMV does not absorb at 594 nm or 646 nm, and the molar extinction coefficient of the virus at 260 nm was reported to be 2.7·10^7^ M^−1^cm^−1^ (5.87 cm^2^ mg^−1^. [42,43]). The molar extinction coefficient of Atto590 at 594 nm is 120,000 M^−1^cm^−1^; the absorbance of Atto590 at 260 nm is relatively high with an extinction coefficient of 46,800 M^−1^cm^−1^. The molar extinction coefficient of Atto647N at 646 nm is 150,000 M^−1^cm^−1^; the absorbance of Atto647N at 260 nm is low with an extinction coefficient of 6000 M^−1^cm^−1^. The fluorophore absorbance at 260 nm was taken into consideration for calculating the DOL. For CCMV labeled with only Atto647N, a DOL of 43 fluorophores/virus was obtained. For CCMV labeled with a FRET pair, DOLs of 8, 14, 21 and 26 fluorophores/virus were obtained with donor to acceptor ratio of 5:3, 10:4, 14:7 and 21:5, respectively.

Emission spectra were recorded using λ_excitation_ = 630 nm and λ_excitation_ = 590 nm to excite Atto647N and Atto590, respectively. Fluorescence decays were recorded using a 590 nm fiber-coupled pulsed laser (Fianium Supercontinuum, SC400-pp, Limoges, France) light source. The fluorescence decays were analyzed using the DAS6 Decay Analysis software (HORIBA Scientific, Limoges, France).

### 3.3. Virus Capsid Disassembly

The surfactant sodium dodecyl sulfate (SDS) was used to disassemble the virus capsids [21,22]. The disassembly of labeled CCMV viruses was monitored in 50 mM phosphate buffer at SDS concentrations up to 1.7 mM. After 30 min incubation of the (labeled) CCMV with SDS, the different spectroscopic experiments were performed.

## Data Availability

The data presented in this study are available on request from the corresponding author.

## Sample Availability

Not available.
